# Replacing sedentary time with physical activity or sleep: effects on cancer-related cognitive impairment in breast cancer survivors

**DOI:** 10.1186/s12885-018-4603-3

**Published:** 2018-06-25

**Authors:** Diane K. Ehlers, Jason Fanning, Elizabeth A. Salerno, Susan Aguiñaga, Josh Cosman, Joan Severson, Arthur F. Kramer, Edward McAuley

**Affiliations:** 1 University of South Carolina, Columbia, SC 29208 USA; 2 Wake Forest School of Medicine, Winston-Salem, NC USA; 3 National Cancer Institute, Rockville, MD USA; 4 University of Illinois at Urbana-Champaign, Urbana, IL USA; 5 Pfizer Incorporated, Cambridge, MA USA; 6 Digital Artefacts, Iowa City, IA USA; 7 Northeastern University, Boston, MA USA

## Abstract

**Background:**

Evidence suggests reallocating daily sedentary time to physical activity or sleep confers important health benefits in cancer survivors. Despite emerging research suggesting physical activity as a treatment for cancer-related cognitive impairment (CRCI), little is known about the interactive effects of behaviors across the 24-h period. The present purpose was to examine the cognitive effects of reallocating sedentary time to light-intensity physical activity, moderate-to-vigorous physical activity (MVPA), or sleep in breast cancer survivors.

**Methods:**

Breast cancer survivors (*N* = 271, *M*age = 57.81 ± 9.50 years) completed iPad-based questionnaires and cognitive tasks assessing demographics, health history, executive function, and processing speed (Task-Switch, Trail Making). Participants wore an accelerometer for seven consecutive days to measure their sedentary, physical activity, and sleep behaviors. Single effects (each behavior individually) and partition (controlling for other behaviors) models were used to examine associations among behaviors and cognitive performance. Isotemporal substitution models were used to test the cognitive effects of substituting 30 min of sedentary time with 30 min of light-intensity activity, MVPA, and sleep.

**Results:**

MVPA was associated with faster Task-switch reaction time in the partition models (stay: *B* = − 35.31, *p* = 0.02; switch: *B* = − 48.24, *p* = 0.004). Replacing 30 min of sedentary time with 30 min of MVPA yielded faster reaction times on Task-Switch stay (*B* = − 29.37, *p* = 0.04) and switch (*B* = − 39.49, *p* = 0.02) trials. In Trails A single effects models, sedentary behavior was associated with faster completion (*B* = − 0.97, *p* = 0.03) and light-intensity activity with slower completion (*B* = 1.25, *p* = 0.006). No single effects were observed relative to Trails B completion (all *p* > 0.05). Only the effect of MVPA was significant in the partition models (Trails A: *B* = − 3.55, *p* = 0.03; Trails B: *B* = − 4.46, *p* = 0.049). Replacing sedentary time with light-intensity activity was associated with slower Trails A (*B* = 1.55 *p* = 0.002) and Trails B (*B* = 1.69, *p* = 0.02) completion. Replacing light activity with MVPA yielded faster Trails A (*B* = − 4.35, *p* = 0.02) and Trails B (*B* = − 5.23, *p* = 0.03) completion.

**Conclusions:**

Findings support previous research suggesting MVPA may be needed to improve cognitive function in breast cancer survivors. Trails findings underscore the need to dissect sedentary contexts to better understand the impact of daily behavioral patterns on CRCI. Additional research investigating the cognitive impacts of behaviors across the 24-h period is warranted.

**Trial registration:**

This study is registered with United States ClinicalTrials.gov (NCT02523677; 8/14/2015).

**Electronic supplementary material:**

The online version of this article (10.1186/s12885-018-4603-3) contains supplementary material, which is available to authorized users.

## Background

Improvements in cancer detection and treatment have resulted in a burgeoning population of cancer survivors in the United States (US). While these improvements represent an important advancement in cancer care, researchers and clinicians face new health challenges associated with cancer survivorship and aging. Breast cancer survivors (BCS) comprise one of the largest survivor populations, with over 3 million living in the US today [1]. Unfortunately, BCS report a number of physical, emotional, and cognitive sequelae related to their cancer diagnosis and treatment. Cognitive deficits due to cancer have increasingly been recognized as a clinical research priority, with some studies suggesting up to 83% of BCS report cognitive impairment after diagnosis [2]. These impairments can be intense, disruptive, and last for durations up to 20 years after treatment ends [3]. The increasing prevalence of cancer-related cognitive impairment (CRCI), as a result of the rapidly growing population of adults at the intersection of cancer-related and age-related cognitive decline, indicate a critical need to investigate potential treatments for CRCI [4, 5].

While a number of treatment modalities have been identified [6], recent studies provide compelling evidence in support of physical activity for mitigating cognitive impairments in cancer survivors [7–9]. Ehlers and colleagues [10] found that more daily minutes of objectively measured moderate-to-vigorous physical activity (MVPA) were associated with better performance across seven tasks of executive function and working memory in a sample of 299 BCS. Marinac and colleagues [11] observed similar relationships between MVPA and processing speed in a sample of 136 postmenopausal BCS. Experimental studies provide further support of these observational findings. Zimmer and colleagues (2016), in the only review of these relationships, found that exercise training may be a promising behavioral modality for CRCI; yet, evidence is limited due to few studies in human models and poor study quality. Hartman and colleagues (2018) recently observed improvements in processing speed among BCS enrolled in a 12-week physical activity intervention compared with controls. Unfortunately, BCS spend significantly more time sedentary and less time engaged in physical activity when compared with women not diagnosed with cancer [12–14].

An emerging literature has specifically focused on the deleterious health effects of extended periods of sedentary behavior in the general adult population and cancer survivors [12, 15, 16]. Voss and colleagues [17] argued that even adequate amounts of daily MVPA may not offset the negative impacts of prolonged sitting on brain health and cognitive function. In other words, individuals who meet the federal guidelines for physical activity (≥ 150 min per week of MVPA) [18], but also engage in long bouts of sitting may still be subject to significant health risks. Additionally, a number of studies suggest sleep deprivation may be associated with accelerated cognitive decline across the lifespan [19]. Empirical studies have suggested that reallocating daily sedentary time to MVPA, light activity, or sleep may confer important benefits to physical health, well-being, and cognition in older adults [20–22]. For example, Fanning and colleagues [22], using a statistical estimation technique called isotemporal substitution modeling, observed hypothetical benefits to older adults’ executive function when substituting 30 min of sedentary time with 30 min of MVPA or sleep. As the biological pathway of cancer-related cognitive decline is thought to represent an accelerated and intensified version of age-related cognitive decline, this evidence from the aging literature may be applicable to cancer survivors.

A small number of studies have explored relationships between sedentary time reallocation and health in cancer survivors. However, this research has restricted its focus to health-related quality of life (HRQoL) outcomes and findings have been mixed. For example, Phillips and colleagues [23] and van Roekel and colleagues [24] observed benefits of light-intensity physical activity and MVPA on fatigue and HRQoL in BCS and colorectal cancer survivors, respectively. Trinh and colleagues [25] also found that sedentary behavior in BCS engaging in low amounts of MVPA was associated with higher levels of fatigue, pain, and depression. Similarly, Vallance and colleagues [26], using isotemporal substitution modeling in non-Hodgkin lymphoma survivors observed significant improvements in fatigue and clinically important improvements in HRQoL when substituting sedentary activity with MVPA. As HRQoL outcomes, such as fatigue, are thought to be associated with CRCI [10, 27], studies investigating the effects of sedentary time reallocation on CRCI are warranted.

The pool of time during which one can engage in these behaviors is finite; therefore, engagement in one behavior replaces time spent in another behavior. While MVPA undoubtedly has the greatest health benefits, more research investigating interactive effects of behaviors across the 24-h period (sleep, sedentary time, light-intensity activity, MVPA) on cognitive function in cancer survivors is warranted. Surveillance data suggest BCS may participate in as little 3.7 min of MVPA per day, with MVPA comprising only 1 % of BCS’s daily wake time [12]. As such, exercise prescriptions promoting MVPA may not be the most attractive or accessible to cancer survivors compared to prescriptions promoting lower intensity physical activities [28–30]. Understanding the health benefits of behaviors across the 24-h day may improve the delivery and effectiveness of cancer rehabilitation and ultimately have greater public health impact.

Using isotemporal substitution modeling, the purpose of the present study was to examine the estimated cognitive effects of substituting daily sedentary time with light-intensity physical activity, MVPA, or sleep. We hypothesized that reallocating 30 min of sedentary time per day to 30 min of light-intensity physical activity, MVPA, or sleep would be associated with improved performance on cognitive tasks of speed of processing and executive function.

## Methods

### Participants and procedures

Women aged 21 years and older who had completed treatment for breast cancer and had access to an iPad with iOS 6.1 or later were eligible to participant in the study. Participants were recruited via the Army of Women©, BreastCancerTrials.org, social media, emailed flyers, and word of mouth. The data presented herein represent cross-sectional findings from a subsample of breast cancer survivors enrolled in a larger prospective observational study examining relationships between physical activity and cognitive function (*N* = 300 of 430). Specifically, interested individuals were asked to download a free iPad application (app; Digital Artefacts, Iowa City, IA) [31] designed for this study and including a series of questionnaires and cognitive tasks. All participants provided institutional review board-approved electronic informed consent prior to their participation and were instructed to complete the assessments within 14 days of signing the consent form. The entire questionnaire battery and cognitive battery (including two practice trials) were designed to be completed in approximately 45 min each. Participants were not required to complete all assessments within one sitting, but could complete part of the questionnaire battery or only one cognitive task at each session [10]. All participants were invited to wear the accelerometer; however, this portion of the study was optional. Therefore, the present study included only those women who agreed to wear the accelerometer and had valid physical activity data (*N* = 300). BCS who wore the accelerometer did not differ from those who did not wear the monitor on any demographic or clinical characteristics with the exception of receipt of radiation therapy. Women who wore the accelerometer were more likely to have received radiation.

### Measures

#### Demographic and clinical information

The questionnaire battery included measures asking participants to report their demographic information and breast cancer history. Variables included age, race/ethnicity, marital status, employment status, education, income level, and previous use of cognitive training tools; breast cancer diagnosis date, stage, estrogen receptor, and menopausal status; chemotherapy, radiation therapy, surgery, and adjuvant hormonal therapy history. These questionnaires have been used in our previous research in BCS (Additional file 1) [10].

#### Daily activity and sleep

Participants were mailed an accelerometer (Actigraph GT3X, Pensacola, FL) within approximately 2 months of signing the consent form (mean = 33.6 ± 14.7 days; Range 0–83 days). Any mailing delays were due to accelerometer availability, participant response to invitation, participant travel schedule, or device re-wear. Devices were initialized to capture movement in 1-s epochs. Participants were instructed to wear the device on a waistband on their non-dominant hip during their wake period, move it to their non-dominant wrist using a wrist band immediately before going to bed, and return it to their waist upon wakening. Women who were diagnosed with lymphedema or experienced any discomfort on their non-dominant side were asked to wear it on their dominant wrist during sleep if possible.

Each participant kept a log of their wake and bed times, which were used to filter the data for separate wear time validation and scoring between wake and sleep periods in Actilife Version 6 (Actigraph, Pensacola, FL). After filtering out sleep windows, daytime non-wear periods were defined as the presence of ≥ 60 consecutive “zero” intensity counts. Physical activity data were considered valid if the device was worn at least 10 h during the participant’s waking hours and on at least 4 days [32], and sleep data were considered valid if the device was worn to bed as determined by participant record of use logs and manual inspection of the data. Valid activity data were scored using Freedson cutpoints [33] and are represented as average daily minutes spent in sedentary, light, and moderate-to-vigorous activity. Sleep data were scored using the Sadeh algorithm [34] and are represented as average daily minutes of sleep. Daily time spent sleeping, sedentary, in light-intensity physical activity and in MVPA, in addition to total time (i.e., sleep + sedentary + light + MVPA), were scaled to increments of 30 min for modeling purposes and to aid in interpretability [22, 26, 35, 36].

#### Cognitive function

The cognitive testing module of the mobile app was powered by BrainBaseline© (www.brainbaseline.com; Digital Artefacts, Iowa City, IA), a commercially-available neuropsychological testing platform. BrainBaseline© includes standard, laboratory-based cognitive tasks with high internal consistency and test-retest reliability. Lee and colleagues [31] demonstrated the utility and initial validity of BrainBaseline© in a sample of 15,346 individuals aged 10–60+ years, and the platform has since been used in other studies [10, 37]. Raw data were downloaded from BrainBaseline© and processed in MATLAB version 8.4 (Mathworks, Inc., Natick, MA) to calculate summary scores for each task.

The Task-Switch [38] and Trail Making [39] tasks were used to measure participants’ cognitive functioning (i.e., speed of processing and executive function) in the present study. Task-switch trials began with the presentation of a pink or blue square at the center of the screen, inside of which was a number (1–4 or 6–9). Numbers were presented individually for 2500 milliseconds. When the square was blue, participants were asked to report as quickly as possible whether the number was higher or lower than 5 using one hand. When the square was pink, participants were asked to report as quickly as possible whether the number was odd or even using the other hand. Participants complete 48 trials in which the task switched randomly across trials. Task performance in the present study was measured as accuracy and reaction time on the stay trials (i.e., color presented is same as previous trial) and switch trials (i.e., color presented is different from previous trial) separately. Accuracies and reaction times were recoded as missing if participants did not achieve 50% accuracy on the task [10].

During the Trail Making task, participants used their finger to draw a line between a series of numbers and/or letters in ascending order. Trails A targets were comprised of numbers only (i.e., 1, 2, 3, etc.). During Trails B trials, participants alternated between numbers and letters in ascending order (i.e., 1, A, 2, B, etc.). Participants were instructed to finish each task as quickly as possible. Task performance in the present study was measured as the time to complete each trail. Task-Switch took about 5 min to complete, and Trail Making (both Trails A and B) took about 3 min to complete with practices. Participants were required to complete the cognitive tasks in the order in which they appeared in the app, with Trail Making immediately following the Task-Switch task.

### Data analysis

Linear regression modeling was used to examine relationships among the predictor variables (sedentary time, light activity, MVPA, and sleep duration) and cognitive outcomes (accuracy and reaction time on Task-Switch and overall time on Trails A and B). Visual inspection of the partial regression plots and scatter plots of the studentized residuals against the unstandardized predict values indicated linear relationships between independent (activity behaviors and sleep) and dependent variables (cognitive performance). Inspection of standardized residual histograms and P-P plots indicated the residuals were approximately normal. Independent and dependent variables were Winsorized at 3 standard deviations from the mean due to the presence of a small number of outliers. We ran three regression models for each outcome, including single effects, partition, and isotemporal substitution models [21, 26, 35]. In the single effects model, the effect of each behavioral predictor on cognitive performance was tested without the other behavioral predictors, but was adjusted for total time. In the partition model, the effects of each behavioral predictor were tested while controlling for the other behaviors. In the isotemporal substitution model, the total time variable was included as a predictor, while the variable of reallocation (i.e., sedentary time) was excluded from the model. The coefficients were interpreted as the mean effect of replacing 30 min of sedentary time with 30 min of each respective activity, while holding the other activities constant [35]. Models were adjusted for age, months of adjuvant hormonal therapy, receipt of chemotherapy, and total time the accelerometer was worn [40]. Additional adjustment for stage of diagnosis, months since diagnosis, education level, and menopausal status did not elicit meaningful changes to the results. Statistical significance was tested at an alpha of *p* ≤ 0.05. Results are presented as unstandardized coefficients and 95% confidence intervals (CIs; Tables 2, 3, 4 and 5). All data were analyzed in SPSS 24.

## Results

**Fig. 1:**
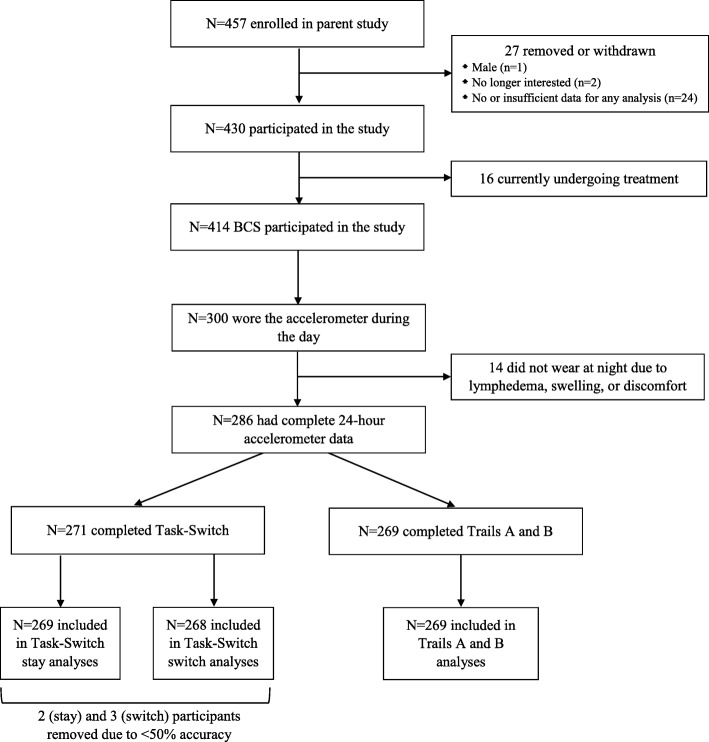
Flow of Participants through the Study

Participants were 271 BCS aged 28–79 years (*M* age = 57.81 ± 9.67; Table 1) drawn from a larger study of 430 women. From the parent study, 300 BCS who had completed primary treatment for breast cancer wore the accelerometer during their wake period and had valid physical activity data. Fourteen participants were missing sleep data due to lymphedema, wrist swelling, or discomfort, leaving 286 women eligible for analysis. Of these, 271 completed the Task-switch task and 269 completed Trails A and B. Finally, two participants had invalid task-switch data on both the stay and switch trials due to accuracies < 50%, and one had invalid switch trial data only. The flow of participants through the present study is detailed in Fig. 1.

**Table 1:** Sample characteristics

	M	±SD^a^
	*n*	(%)
Age (years)	57.81	±9.50
Bachelors Degree	219	(80.8)
Income ≥ $75,000 per year (*n* = 256)	193	(75.4)
Employed full-time	106	(39.1)
Retired	96	(35.4)
White	258	(95.2)
Married	210	(77.5)
Cancer Stage		
0	20	(7.4)
1	110	(40.6)
2	93	(34.3)
3	42	(15.5)
4	6	(2.2)
Months since diagnosis	95.13	±73.30
History of chemotherapy only	42	(15.5)
History of radiation only	51	(18.8)
History of chemotherapy and radiation	150	(55.4)
Hormonal therapy (months)	21.41	±31.17
Body mass index (BMI)	26.74	±5.83
Accelerometer-derived estimates		
Total daily wear time (min)	1322.80	±44.09
Wake-time daily wear (min)^b^	912.38	±61.11
Daily sedentary behavior (min)	600.03	±73.24
Daily light-intensity PA (min)	283.12	±69.65
Daily moderate-to-vigorous PA (min)	29.23	±22.71
Daily sleep duration (min)	411.16	±47.39
Task-Switch		
Stay accuracy (%)	94.07	±10.49
Stay reaction time (ms)	1166.48	±157.62
Switch accuracy (%)	93.46	±10.60
Switch reaction time (ms)	1373.29	±175.64
Trails A time (sec)	53.96	±20.04
Trails B time (sec)	67.67	±28.55

^a^Mean, Standard Deviation

^b^Sum of daily time spent in sedentary, light, and MVPA behaviors

### Task-switch (Tables 2 and 3)

No significant effects of any behaviors on Task-switch stay or switch accuracy were observed across all models (all *p* > 0.05; no table included). In single effects models, MVPA was marginally associated with faster reaction time on stay trials (*B* = − 27.45, *p* = 0.06; Table 2) and significantly associated with fast times on switch trials (*B* = − 38.63, *p* = 0.02; Table 3). The effect of MVPA was significant in both partition models (stay: *B* = − 35.31, *p* = 0.02; switch: *B* = − 48.24, *p* = 0.004). Substituting 30 min of sedentary behavior, light-intensity activity, or sleep with MVPA yielded faster reaction times on stay trials (sedentary: *B* = − 29.37, *p* = 0.04; light: *B* = − 35.03, *p* = 0.03; sleep: *B* = − 30.47, *p* = 0.055) and switch trials (sedentary: *B* = − 39.49, *p* = 0.02; light: *B* = − 41.917, *p* = 0.03; sleep: *B* = − 39.67, *p* = 0.03). Reallocating sedentary behavior to light-intensity activity or sleep was not associated with faster reaction time on either Task-switch outcome (all *p* > 0.21).

**Table 2:** Effects of activity type on task-switch stay reaction times

Activity				
	Sedentary Time	Light Activity	MVPA	Sleep
Single effect	− 1.47 [− 9.62, 6.69]	4.45 [− 3.79, 12.69]	−27.45 [− 55.85, 0.94]	−1.41 [− 13.50, 10.68]
Partition effect	−5.94 [− 18.07, 6.18]	−0.28 [− 12.89, 12.33]	**−35.31 [− 64.98, − 5.64]**	−4.84 [− 19.46, 9.78]
Isotemporal effect				
Replace sedentary time with…	Dropped	5.67 [− 3.36, 14.69]	**−29.37 [− 57.96, − 0.78]**	1.11 [− 12.07, 14.28]
Replace light activity with…	− 5.67 [− 14.69, 3.36]	Dropped	**− 35.03 [− 65.75, − 4.32]**	− 4.56 [− 17.16, 8.04]
Replace MVPA with…	**29.37 [0.78, 57.96]**	**35.03 [4.32, 65.75]**	Dropped	30.47 [− 0.65, 61.59]
Replace sleep with…	−1.11 [− 14.28, 12.07]	4.56 [− 8.04, 17.16]	−30.47 [− 61.59, 0.65]	Dropped

Note. Effects are presented as unstandardized coefficients [95% confidence interval] of Trails completion in *seconds.* Single effects and isotemporal models adjusted for age, months of adjuvant hormonal therapy, receipt of chemotherapy, and total time. Bold indicates *p* < 0.05

**Table 3:** Effects of activity type on task-switch switch reaction times

Activity				
	Sedentary Time	Light Activity	MVPA	Sleep
Single effect	2.06 [−7.04, 11.16]	1.16 [−8.06, 10.37]	**− 38.63 [− 70.18, − 7.07]**	− 0.09 [− 13.59, 13.40]
Partition effect	−8.75 [− 22.26, 4.76]	−6.33 [− 20.38, 7.73]	**− 48.24 [− 81.30, − 15.19]**	−8.58 [− 24.86, 7.71]
Isotemporal effect				
Replace sedentary time with…	Dropped	2.42 [−7.64, 12.48]	**−39.49 [− 71.35, − 7.64]**	0.18 [− 14.51, 14.86]
Replace light activity with…	−2.42 [− 12.48, 7.64]	Dropped	**− 41.91 [− 76.13, − 7.70]**	−2.25 [− 16.29, 11.79]
Replace MVPA with…	**39.49 [7.64, 71.35]**	**41.91 [7.70, 76.13]**	Dropped	**39.67 [4.99, 74.34]**
Replace sleep with…	−0.18 [− 14.86, 14.51]	2.25 [− 11.79, 16.29]	**−39.67 [− 74.34, − 4.99]**	Dropped

Note. Effects are presented as unstandardized coefficients [95% confidence interval] of Trails completion in *seconds.* Single effects and isotemporal models adjusted for age, months of adjuvant hormonal therapy, receipt of chemotherapy, and total time. Bold indicates *p* < 0.05

### Trails A total time (Table 4)

In the single effects model, sedentary time was significantly associated with faster Trails A completion (*B* = − 0.97, *p* = 0.03; Table 4), while minutes of light-intensity activity were associated with slower Trails A completion (*B* = 1.25, *p* = 0.006). MVPA and sleep were not significantly associated with Trails A completion in the single effects model (*p* = 0.15, 0.81). However, in the partition model, only MVPA was associated with Trails A completion time (*B* = − 3.55, *p* = 0.03). Replacing 30 min of sedentary behavior with 30 min of light-intensity activity was associated with slower Trails A completion (*B* = 1.55, *p* = 0.002). Substituting sedentary behavior with MVPA or sleep was not significantly associated with changes in Trails A time (*p* = 0.08, 0.33). When light-intensity activity was replaced with sedentary behavior or MVPA, Trails A times were estimated to be faster (sedentary: *B* = − 1.55, *p* = 0.002; MVPA: *B* = − 4.35, *p* = 0.01).

**Table 4:** Effects of activity type on trails A completion

Activity				
	Sedentary Time	Light Activity	MVPA	Sleep
Single effect	**−0.97 [−1.85, − 0.08]**	**1.25 [0.36, 2.14]**	−2.29 [− 5.40, 0.83]	−0.16 [− 1.48, 1.16]
Partition effect	− 0.75 [− 2.05, 0.55]	0.80 [− 0.55, 2.14]	**−3.55 [− 6.76, − 0.34]**	− 0.36 [− 1.61, 1.54]
Isotemporal effect				
Replace sedentary time with…	Dropped	**1.55 [0.57, 2.52]**	− 2.80 [− 5.89, 0.29]	0.71 [− 0.72, 2.14]
Replace light activity with…	**−1.55 [− 2.52, − 0.57]**	Dropped	**−4.35 [− 7.68, − 1.02]**	− 0.83 [− 2.19, 0.53]
Replace MVPA with…	2.80 [−0.29, 5.89]	**4.35 [1.02, 7.68]**	Dropped	**3.51 [0.13, 6.89]**
Replace sleep with…	−0.71 [− 2.14, 0.72]	0.83 [− 0.53, 2.19]	**−3.51 [− 6.89, − 0.13]**	Dropped

Note. Effects are presented as unstandardized coefficients [95% confidence interval] of Trails completion in *seconds.* Single effects and isotemporal models adjusted for age, months of adjuvant hormonal therapy, receipt of chemotherapy, and total time. Bold indicates *p* < 0.05

### Trails B total time (Table 5)

The single effects of sedentary time, MVPA, light-intensity activity, and sleep were not associated with Trails B completion time (all *p* > 0.05; Table 5). In the partition model, MVPA was significantly associated with faster Trails B time (*B* = − 4.46, *p* = 0.049). In the isotemporal substitution model, replacing 30 min of sedentary time with 30 min of light-intensity activity or sleep was significantly or marginally associated with slower Trails B completion (light: *B* = 1.69, *p* = 0.02; sleep: *B* = 1.91, *p* = 0.058). Replacing 30 min of sedentary behavior with 30 min of MVPA did not influence Trails B completion (*p* = 0.10). However, replacing light-intensity activity with MVPA was associated with faster Trails B completion (*B* = − 5.23, *p* = 0.03). A significant association was also observed when MVPA replaced sleep (*B* = − 5.45, *p* = 0.02).

**Table 5:** Effects of Activity Type on Trails B Completion

Activity				
	Sedentary Time	Light Activity	MVPA	Sleep
Single effect	−1.19 [− 2.41, 0.03]	1.02 [−0.21, 2.26]	−3.16 [− 7.43, 1.12]	0.97 [− 0.85, 2.79]
Partition effect	−0.92 [− 2.71, 0.87]	0.77 [− 1.10, 2.63]	**−4.46 [− 8.90, − 0.03]**	0.99 [−1.19, 3.17]
Isotemporal effect				
Replace sedentary time with…	Dropped	**1.69 [0.33, 3.04]**	− 3.54 [− 7.81, 0.72]	1.91 [− 0.07, 3.89]
Replace light activity with…	**−1.69 [− 3.04, − 0.33]**	Dropped	**− 5.23 [− 9.83, − 0.63]**	0.22 [− 1.65, 2.10]
Replace MVPA with…	3.54 [− 0.72, 7.81]	**5.23 [0.63, 9.83]**	Dropped	**5.45 [0.78, 10.12]**
Replace sleep with…	− 1.91 [− 3.89, 0.07]	−0.22 [− 2.10, 1.65]	**−5.45 [− 10.12, − 0.78]**	Dropped

Note. Effects are presented as unstandardized coefficients [95% confidence interval] of Trails completion in *seconds.* Single effects and isotemporal models adjusted for age, months of adjuvant hormonal therapy, receipt of chemotherapy, and total time. Bold indicates *p* < 0.05

## Discussion

The purpose of this study was to investigate the estimated cognitive effects of reallocating daily sedentary behavior to light-intensity physical activity, MVPA, and sleep in BCS. A major strength of this study is the use of objective measures of physical activity, sleep, and cognitive function in a large sample of BCS. Contrary to our hypotheses, findings suggest the benefits of sedentary time replacement to speed of processing and executive function may be restricted to lifestyle behaviors of at least a moderate intensity. Only MVPA was associated with faster task-switch performance in the sedentary time substitution models, while replacing sedentary time with light-intensity activity yielded slower performance on the Trails tasks. Replacing light-intensity activity with MVPA resulted in faster performance on all tasks. These findings are generally consistent with previous studies and may pose important challenges to the design of interventions aimed at mitigating cognitive impairments in BCS.

In one of the few studies also examining the hypothetical effects of sedentary time replacement on cognitive functioning, Fanning and colleagues [22] observed significant improvements in older adults’ self-regulatory behaviors and performance on executive function tasks when 30 min of sedentary behavior was substituted with 30 min of MVPA. Further, similar to the present study, replacing sedentary time with light-intensity activity did not lead to improved cognitive performance. The aging literature in general provides strong and consistent evidence in support of moderate-to-vigorous aerobic exercise training for improving cognitive functioning and brain health in older adults [41]. These associations have been replicated in studies of physical activity and CRCI [7, 8, 10, 11, 42]. However, none have investigated the cognitive benefits of MVPA in conjunction with, independent of, or in replacement of other activity behaviors in cancer survivors. Our findings fill this knowledge gap with preliminary evidence relative to MVPA, speed of processing, and executive function.

A number of studies have documented beneficial effects of replacing sedentary time with light-intensity activity on cardiometabolic health, body composition, physical function, and psychosocial well-being in older adults and cancer survivors [20, 21, 43–45]. Vallance and colleagues [26], on the other hand, found that only MVPA in 10-min bouts or more were associated with improved health outcomes (i.e., fatigue, HRQoL) in a sample of 149 non-Hodgkin lymphoma survivors. Fatigue is a known correlate of CRCI [27] and has been documented as a potential mediator of the relationship between MVPA and CRCI [10, 46]. Such findings, in combination with those of the present study, may have important implications for the promotion of health-enhancing physical activity in cancer survivors. While sedentary time replacement strategies generally result in increases in light-intensity activity, clinicians may consider approaches that promote MVPA when targeting survivors’ cognitive functioning. Certainly the suggestion is not to eliminate whole day approaches to health behavior promotion [47]. However, evidence in support of physical activities of at least a moderate intensity for the improvement of cognitive function is compelling.

Of further interest was the relationship observed between sedentary behavior and light-intensity physical activity in the Trails A and B substitution models. Both Trails A and B completion were estimated to be slower when 30 min of sedentary behavior was replaced with 30 min of light-intensity activity. Despite a plethora of evidence relative to MVPA’s influence on cognitive function and brain health across populations, less is known about the influences of sedentary behavior and light-intensity physical activity [17]. Our study is among only a few, to our knowledge, to examine associations between light-intensity physical activity and cognition, and present findings are contrary to those of previous studies. For example, Buchman et al. [48, 49] found that total daily physical activity (actigraphy derived counts) and intensity of physical activity (counts per hour) were associated with greater cognitive function and lower risk of Alzheimer’s disease in older adults. While these data suggest that physical activity, regardless of intensity level, has cognitive benefits, the authors did not specifically isolate non-exercise or low-intensity activity. However, more recently, Varma and colleagues [50] linked low-intensity walking activity, independent of MVPA and self-reported exercise, with hippocampal volume in older adults. It is possible that the effects of light-intensity activity on cognitive processes may be specific to certain domains. The hippocampus is known to control memory processes, while the present study included measures of processing speed and executive function. Further research is warranted to understand how daily behavioral profiles influence cognitive function across domains known to be amenable to physical activity and sleep.

The effects of physical activity on cognitive function are indeed dose-dependent, with MVPA eliciting the greatest behavioral response in cognition [17, 48]. Our findings in support of MVPA are not unlike those of previous studies focusing on cognitive function [22] or other health outcomes (e.g., fatigue, quality of life) in cancer survivors [26]. However, as these previous studies observed null effects related to light-intensity physical activity, it remains unclear why reallocating sedentary behaviors to light-intensity activity yielded slower performance on both Trails A and B in the present study. It is possible that higher doses of physical activity may have been required to elicit significant cognitive responses in our sample of active and higher functioning BCS. Our eligibility criteria did not exclude physically active BCS, as reflected in the mean MVPA of 30 min per day. Further, although normative cognitive data for cancer survivors are not currently available, cognitive functioning among participants in the present sample may have been comparable to or even higher than that of the general population of similarly-aged adults [31]. While the present study provides evidence in support of an MVPA prescription for improved cognitive health in BCS, the counterintuitive effects of light-intensity activity warrant further investigation. As MVPA comprises only a small proportion of daily behavior (Table 1), experimental studies specifically testing the effects of light-intensity activities on cognitive function and other health outcomes in BCS may provide the most insightful information [51].

Contrary to our hypothesis, little association between sleep and cognitive performance was observed. Evidence in support of sleep’s benefits to cognitive functioning are unequivocal in the general adult population [52, 53]. While no consensus has been reached on the amount of sleep required to optimize cognitive functioning, studies have suggested that habitual sleep durations of fewer than 6 h or more than 9–9.5 h are related to increased cognitive impairment [54]. In the present study, the vast majority of participants had sleep durations of 6–9 h per night (82.0%), indicating generally sufficient sleep across the sample. While sleep disturbances are thought to be more prevalent in BCS when compared with non-cancer populations, Budhrani and colleagues [55] demonstrated in a recent review that total sleep time may not differ between BCS and non-cancer adults. Additionally, other sleep metrics may better explain the influence of sleep on cognitive function across ages and populations [19]. In future studies exploring the effects of sleep on CRCI, investigations of other sleep quality outcomes, such as wakefulness after sleep onset, sleep onset latency, and daytime dysfunction, may be more informative than sleep duration alone.

### Limitations

This study had a number of strengths, including enrollment of a national sample of BCS, objective measures of daytime and sleep behaviors via actigraphy, and objective measures of cognitive functioning. Despite these strengths, this study also had limitations. First, participants represented a homogeneous population of Caucasian, well-educated, and affluent breast cancer survivors. Therefore, generalizability of the results to other populations of breast cancer survivors is limited. Additionally, several participants reported daytime naps on their accelerometer log. We did not ask participants to record such information and, therefore, did not remove any reported daytime nap periods from activity calculations. As such, bouts of daytime sleep were most likely categorized as sedentary time or non-wear. The effects of sleep on health are generally distinct from sedentary behavior [21]. Further, napping has been associated with improved cognitive function; yet, prolonged napping may also be an indicator of underlying health conditions [56]. Therefore, we not only were unable to test the effects of daytime sleep on cognitive function, but napping, if widespread across the sample, may also have inhibited our ability to full test the effects of sedentary behavior on cognitive function. Because sleep dysfunction is common among cancer survivors [55], efforts to understand health conditions associated with daytime sleep in cancer survivors and the effects of napping on health outcomes, such as cognitive function, are needed.

Similarly, our objective measure did not provide us with any contextual information about sedentary behaviors. Television viewing, for example, has consistently been associated with poorer health outcomes in older adults, while social and cognitive sitting activities, such as talking with friends, reading, or completing a puzzle, may have neuroprotective health effects [57–59]. Further research dissecting daily sedentary behavior among BCS may help us to better explain the effects of light-intensity activity observed in the present study. Finally, causal associations among variables cannot be discerned in the present study due to the cross-sectional design and hypothetical modeling of sedentary time replacement. Prospective and experimental studies are needed to further test interactions among behaviors across the 24-h period.

## Conclusions

Isotemporal substitution models in which sedentary behavior was replaced with physical activity yielded improvements in BCS’s performance on cognitive tasks measuring speed of processing and executive function. More importantly, these improvements were dose-dependent, with MVPA conferring the most benefit and light-intensity activity resulting in declined performance in some models. In fact, reallocating MVPA to sedentary activity, light-intensity activity, or sleep was consistently associated with poorer cognitive performance. Further testing of interactions among sedentary behavior, light-intensity physical activity, MVPA, and sleep on CRCI is needed to inform the design of cancer rehabilitation strategies targeting cognitive function in cancer survivors.

## Additional file

Additional file 1: Supplementary File (Additional Questionnaires). (PDF 78 kb)

## Abbreviations

BCS: Breast cancer survivors
CRCI: Cancer-related cognitive impairment
HRQoL: Health-related quality of life
MVPA: Moderate-to-vigorous physical activity
US: United States
