# Supplementary material for: Replacing sedentary time with physical activity or sleep: effects on cancer-related cognitive impairment in breast cancer survivors
Source: BMC Cancer. 2018 Jun 25;18:685. doi: 10.1186/s12885-018-4603-3 (PMC6019533; doi:10.1186/s12885-018-4603-3)
Supplement: Supplementary file 1 — Supplementary File (Additional Questionnaires). (PDF 78 kb) [file 12885_2018_4603_MOESM1_ESM.pdf]

ReFLECT Study

Instructions:

Thank you for agreeing to participate in the study, **Re**lationships among Cognitive **F**unction, **L**ifestyle, and **E**xercise after **C**ancer **T**reatment (ReFLECT). This questionnaire will ask you a series of questions about yourself. There are no right or wrong answers and all we ask is that you provide responses that are as honest and accurate as possible. All responses are completely confidential and will not be used in any way that could link your responses to you.

Exercise Psychology Laboratory

ILLINOIS
UNIVERSITY OF ILLINOIS AT URBANA-CHAMPAIGN

Please complete the following information about yourself. The information you provide will be used for research purposes only and will be held in **strictest confidence**.

1. What is your current Marital Status? (circle one)
   Married
   Partnered/Significant Other
   Single
   Divorced/Separated
   Widowed

2. What is your **current** Age? ___________

3. Number of Children ___________

4. What is your current Employment Status? (please select only one)
   _____Full time – working at least 35 hours/week
   _____Part time – working less than 35 hours/week
   _____Retired, working part-time
   _____Retired, not working at all
   _____Laid off or unemployed
   _____Full time homemaker
   _____Other, Specify: ____

5. If you are working, what is your Present Occupation (the one you work most hours per week)?

   ____

6. Years in present occupation:____________ years

7. If you are employed, how many days of work have you missed in the past month because you were sick?
   ____

8. Race (circle one)
   American Indian/Alaska Native
   Asian
   Native Hawaiian or Other Pacific Islander
   Black or African American
   White
   More Than One Race
   Unknown or Not Reported

9. Ethnicity (circle one)
   Hispanic or Latino
   Not Hispanic or Latino

10. Education (Circle highest level attained)
    1. Less than 9th grade
    2. 9th grade (Jr. High)
    3. Partial High School
    4. High School Graduate
    5. 1-3 years of College or 2 yr College/Vocational/Technical school graduate
    6. College/University Graduate
    7. Masters Degree
    8. PhD or Equivalent

11. Annual Household Income (circle one)
    1. $25,000 to $34,999
    2. $35,000 to $49,999
    3. $50,000 to $74,999
    4. $75,000 to $99,999
    5. $100,000 to $149,999
    6. $150,000 or more

12. Do you have a psychological or social characteristic that may prevent you from accurately answering the study questions, completing the cognitive tests, or using the activity monitor (if applicable)? Please choose one.

    Yes
    No

13. Have you used any cognitive training or assessment tools (e.g., BrainBaseline, Lumosity, etc.)?

    Yes
    No

    If yes, what tool(s) have you used?

Please answer the following questions about your breast cancer history.

1. When did you receive your first diagnosis of breast cancer?	________ Month ___________ Year
2. What was your age at diagnosis?	___________
3. What was the stage of your breast cancer?	☐ 1 ☐ 2 ☐ 3 ☐ 4 ☐ Don't know
4. Was your breast cancer estrogen receptor positive?	☐ Yes ☐ No ☐ Don't Know
5. Are you currently receiving chemotherapy for your breast cancer?	☐ Yes ☐ No
6. Have you ever received chemotherapy for breast cancer?	☐ Yes ☐ No
a. If yes, for how many months?	___________
b. Months since last dose of chemotherapy	___________
7. Are you currently receiving radiation therapy for your breast cancer?	☐ Yes ☐ No
8. Have you ever received radiation therapy for your breast cancer?	☐ Yes ☐ No
a. If yes, for how many months?	___________
b. Months since last dose of radiation therapy	___________
9. Did you have surgery for breast cancer?	☐ Yes ☐ No
a. If yes, what surgery was done? ____	
b. Months since your surgery for breast cancer	___________
10. Which of the following medications are you currently taking?	
☐ anastrozole (Arimidex®)	☐ tamoxifen (Nolvadex®)
☐ letrazole (Femara®)	☐ toremifene (Fareston®)
☐ exemestane (Aromasan®)	☐ raloxifene (Evista®)
☐ fulvestrant (Faslodex®)	☐ Not taking any of these
a. Length of time you have been taking above medication	___________
11. Have you experienced menopause?	☐ Yes ☐ No ☐ Don't know
12. Which of the following describes your menopausal status at the time of diagnosis?	
☐ Pre-menopausal ☐ Post-menopausal ☐ Not sure	

13. Have you been diagnosed with a breast cancer recurrence? (By recurrence we mean the breast cancer coming back in the same breast or a new breast cancer in either breast)

☐ Yes ☐ No ☐ Don't know

a. If yes, how many times ___________

b. If yes, when? _________ _________ _________ _________
Month Year Month Year

c. What was the stage of your diagnosis? ☐ 1 ☐ 2 ☐ 3 ☐ 4 ☐ Don't know

14. Have you ever been diagnosed with any other type of cancer?

☐ Yes ☐ No ☐ Don't know

a. If yes, what type____

b. If yes, when? _________ _________ _________ _________
Month Year Month Year

General Health History

Has your doctor diagnosed you with any of the following conditions?

15. Arthritis (rheumatoid and/or osteoarthritis)	☐ Yes	☐ No
16. Osteoporosis	☐ Yes	☐ No
17. Asthma	☐ Yes	☐ No
18. Chronic obstructive pulmonary disease (COPD), acquired respiratory distress syndrome (ARDS) or emphysema	☐ Yes	☐ No
19. Angina	☐ Yes	☐ No
20. Congestive heart failure (or heart disease)	☐ Yes	☐ No
21. Heart attack (myocardial infarct)	☐ Yes	☐ No
22. Neurological disease (such as Multiple Sclerosis or Parkinson's)	☐ Yes	☐ No
23. Stroke or TIA	☐ Yes	☐ No
24. Dementia or organic brain syndrome	☐ Yes	☐ No
25. Peripheral vascular disease (for example blockages in the arteries of your neck, arms and/or legs	☐ Yes	☐ No

26. Diabetes type I or II ☐ Yes ☐ No

27. Upper gastrointestinal disease (ulcer, hiatal hernia, reflux) ☐ Yes ☐ No

28. Tremors ☐ Yes ☐ No

If yes, which part(s) of your body are affected by tremors (e.g., hands, arms, face, voice)?

____

29. Depression ☐ Yes ☐ No

30. Anxiety or panic disorders ☐ Yes ☐ No

31. Visual impairment (such as cataracts, glaucoma, macular degeneration) ☐ Yes ☐ No

32. Hearing impairment (very hard of hearing, even with hearing aids) ☐ Yes ☐ No

33. Degenerative disc disease (back disease, spinal stenosis, or severe chronic back pain) ☐ Yes ☐ No

34. Obesity ☐ Yes ☐ No

35. What is your current weight in pounds? ______________

36. What is your current height in inches? ______________

37. Which of the following is true regarding your current weight in comparison to your pre-cancer weight?

I weigh less now. I weight about the same. I weight more now.

38. Are you satisfied with your current weight?

Yes No

a. If no, how much weight would you like to gain or lose?

Gain (+) ________ pounds Lose (-) ________pounds Neither

39. How many cups of any caffeinated beverage do you drink daily (soft drinks, coffee, tea, etc.)?

____

40. Do you currently smoke?

Yes No

a. If yes, how many packs a day do you smoke? _________

41. Did you smoke previously?

Yes No

a. If you are a former smoker, how many years has it been since you quit? ________

42. Do you drink alcohol?

Yes No

a. If yes, how many days per week do you drink alcohol? _____________

b. How many drinks containing alcohol do you have on a typical day when you are drinking? ____________
